# Social processes in academic-community partnership in health care. A grounded theory study

**DOI:** 10.1186/s12912-021-00784-z

**Published:** 2021-12-23

**Authors:** Susanna Pusa, Susanne Lind, Marie Häggström

**Affiliations:** 1 Institution of Health Care Sciences, Palliative Research Centre Ersta Sköndal Bräcke University College, Ersta Sköndal Bräcke högskola, Box 11189, 100 61 Stockholm, Sweden; 2 Institution of Nursing Sciences, Mid Sweden University, Mittuniversitetet. OMV, 851 70 Sundsvall, Sweden

**Keywords:** Community-based participatory research, Cooperative behavior, Grounded theory, Implementation science, Intersectoral collaboration, Nursing care, Palliative care, Public-private sector partnership, Qualitative research, Social interaction

## Abstract

**Background:**

International and national guidelines state that palliative care should be offered to everyone who needs it. To promote the implementation of palliative care in nursing homes, a partnership collaboration was initiated with the goal of implementing high quality palliative care. The partnership consisted of three partner groups: a project group from a non-profit organisation providing health care, managers at the nursing homes and an academic partner. The aim was to explore the social processes within academic-community partnership in a collaboration project.

**Methods:**

Digital focus group discussions were conducted with 16 participants, representing all three partner groups. One individual digital interview was also carried out. A constructivist perspective of a grounded theory approach was used for data analysis.

**Results:**

The core category, partnership positioning, covers the social processes of the academic-community partnership in a collaboration project to implement and evaluate health-promoting interventions in clinical health care. The core category was found to have four categories: Pre-positioning, Co-positioning, Re-positioning and GoOn-positioning. The process of partnership positioning is conceptualised in a model.

**Conclusions:**

Our findings indicate that a new partnership in an implementation project needs holistic, systemic thinking. To enhance implementation in a collaborative project involving different professionals and actors a plan is required to facilitate positioning activities. The process, the roles and the components need to be clearly defined and documented, and the management of a system requires knowledge of the interrelationships between all the components within the system. The development of a conceptual model of Partnership Positioning contributes to knowledge concerning the social dynamic processes which can be applied to support future academic-community collaboration and/or implementation projects.

**Trial registration:**

Not applicable. The present study has not been considered as a clinical trial.

## Background

Quality improvements need to be constantly performed in order to ensure that the health care that is provided is evidence-based, safe and in accordance with the recommendations of the Swedish National Board of Health and Welfare and other healthcare documents, such as national clinical practice guidelines [1]. During the past decades, knowledge in the field of palliative care has been growing. According to the World Health Organization, palliative care is expected to be offered to every person who needs it, regardless of underlying cause, age or place of care [2]. Further, the International Association for Hospice and Palliative Care emphasises that palliative care is for persons with serious suffering due to severe diseases and aims for an improvement in quality of life of patients and their families [3]. The concept of palliative care includes consideration of four core components: symptom relief, teamwork, communication and relationships, and the support of next of kin [4]. This is in line with the description of nurses’ activities by the International Council of Nurses [5]. The wide range of activities in nursing, both in general and specifically in palliative care, contributes to the view that nursing is a complex activity [6]. A high proportion of deaths in Sweden occur in nursing homes [7]. This indicates that good knowledge of palliative care among healthcare professionals is of importance to ensure that persons in need of palliative care experience as good a quality of life as possible. Several governing documents have been published to support the implementation and development of palliative care in Sweden.

The implementation of palliative care can be considered to be a complex intervention due to several factors. The implementation object and the context, for example palliative care in elderly care, can be perceived as topics that are uncomfortable to talk about, which may affect the fidelity of the implementation (i.e. whether the intervention was delivered as intended) [8]. Further, the design of an implementation strategy and how it is delivered is of importance for the outcome of the intervention. According to Campbell [9], interventions directed at healthcare professionals’ behaviour and containing several components should be considered complex interventions.

When implementing health promoting interventions in clinical care, the evaluation often focuses on clinical treatment outcomes such as patient reported outcomes. However, it is of value to conduct process evaluations to assess and understand an implementation process [10]. Process evaluations should be performed prior to drawing conclusions regarding the effectiveness of an intervention [10] in order to enhance the understanding of how and why clinical outcomes could or could not be reached [11]. Proctor et al. [10] state that if an intervention is not well implemented, it will not be effective. One aspect regarding the process of implementation of an intervention is the collaboration between the partners involved in the project [12].

Collaboration between academics and society, through a partnership, can offer various benefits and support successful implementation [13]. There are several existing definitions of partnership; for instance, in Dámour et al.’s [14] review the definition of partnership is “two or more actors join [ed] in a collaborative undertaking (or a set of common goals and specific outcomes) characterised by a collegial-like relationship that is authentic and constructive”. Huang et al. [15] conceptualise partnership as “a broader umbrella term that includes engagement and collaboration. Partnerships can occur in multiple forms and at different levels”. For the purpose of this study, we define partnership as a contextually relevant collaboration comprising all types of collaboration (e.g. relationships, co-operation and alliances) within and between organisations involved in the overall project to improve palliative care.

A partnership consisting of a community healthcare centre and academic partner/s has the advantage of being able to both promote community health and utilise health research, since the partners each have unique knowledge and expertise [16, 17]. However, the skills needed to construct, manage and sustain partnerships are critical features. Thus, on the one hand, collaborative approaches offer opportunities and, on the other hand, challenges [18]. Furthermore, the complexity of the healthcare system [18] and the complexity of the intervention being implemented [19] should be noted. Taken together, this is all of value to better understand the phenomenon of the dynamic process in partnership.

## Method

### Purpose

The purpose was to explore the social processes within academic-community partnership in a collaboration project.

### Design

The study has an explorative design; a focus group discussion method was used for data gathering and a constructivist grounded theory approach was used for data analysis.

### The setting

In Sweden, the responsibility for health care is divided between the state, county councils and local municipalities. Sweden’s universal health system is nationally regulated and locally administrated. The Ministry of Health and Social Affairs sets the overall health policy while the regions and municipalities deliver health care. The funding comes from regional- and municipality-level taxes and from grants provided by central gouverment. Nursing homes in Sweden are guided by legislation on medical care and social services. The municipality is responsible for managing nursing homes and the care provided can also be performed on behalf of the municipality by private companies, i.e. non-profit or for profit organisations.

The present study is part of a larger project with the overall aim of investigating the outcomes of an educational intervention concerning the general palliative care of older people in nursing homes in Sweden. The project is a collaboration between (1) a project group consisting of a non-profit organisation providing health care in Sweden, (2) managers at the nursing homes and (3) an academic partner (i.e. a research group at a university). Thus, the focus for the partnership collaboration was an educational intervention in general palliative care. The educational intervention was developed by the non-profit organisation in collaboration with a foundation. The content is based on the Swedish National Board of Health and Welfare’s concept of good palliative care at the end of life, including the four core components: communication, symptom relief, teamwork and support of next of kin.

The educational intervention program consists of an existing and established web-based education as well as educational seminars for the staff (managers, registered nurses, enrolled nurses, care assistants, physiotherapists and occupational therapists). The role of the managers in the project and the partnership is to support the clinical implementation in the nursing homes. The academic partners are all registered nurses and teachers in higher education. Their function is to explore and evaluate the outcomes connected to the educational intervention and the implementation process. Consequently, the focus of the present study is not on the educational intervention itself but instead on the social processes within the partnership.

### Data collection

A semi-structured interview guide was designed in accordance with the study purpose and objectives after a review of the literature (e.g. [15, 20]). The interview guide comprised three overarching discussion topics: partnership composition, partnership function and group processes. The questions oscillated between the individual’s own role and the collaboration within and between the partner groups. The focus was on the participants’ perceptions, experiences and suggestions for improvement.

Data collection was carried out through four digital focus group discussions via Microsoft TEAMS with four persons in each group, and one individual interview. Since the focus group discussions and the individual interview were conducted online, the participants could choose from where they wanted to attend. The data collection took place between June and September 2020 and the focus group discussions lasted between 67 and 102 min. The interviewers had little or no previous interaction with the participants. During the focus group discussions author SP took the role as moderator and author SL adopted a more reflective role. Ethical considerations included handling possible diversity of opinions or disagreements during the interviews as well reflecting on potential negative consequences for the including participants after the interviews. However, during the focus group interviews, the atmosphere was open and permissive and no disputes occurred.

Written memos were collected during and directly after the focus group discussions. Additionally, discussions and reflections between the two interviewers were held directly after each focus group discussion. The composition of the focus groups was made based on the organisation and employer the participants worked for, i.e. participants from the same partner groups were included in the same focus group discussion.

### Participants

The sampling was purposive to include participants in the collaborating partner groups. The participants’ partner groups consisted of: (1) the project group from the non-profit organisation providing health care in Sweden, including a representative from the foundation that developed the web-based education, (2) the managers at the nursing homes and (3) an academic partner (i.e. a research group at a university). Demographic data were collected regarding age, gender, current employment, number of years working in current employment, and experience and knowledge of palliative care. For an overview of participants’ characteristics, see Table 1.

**Table 1:** Overview of participants’ demographics

Participants	Missing	Age (years)	Sex		Years of service*	Level of education		
			Female	Male		BSc	MSc	PhD
Project group (from the non-profit organisation providing health care)	1	56–61	3	0	5–24	1	2	0
Managers (at the nursing homes)	0	50–61	8	0	1–10	6	2	0
Academic partner (researchers)	0	56–64	5	0	2–11	0	0	5

*in current employment

### Data analysis

A constructivist perspective of grounded theory in accordance with Charmaz’ [21] approach was used for data analysis. As stated by Charmaz, constructivist grounded theory is both inductive and abductive - an iterative process of analysis. This constructivist approach acknowledges that both the data and the analysis are interactively constructive [21].

Different steps were followed during the analysis. After the audio-recorded focus group discussions and the individual interview were transcribed verbatim, the text was imported into the software program MaxQDA 2020 where the initial coding started. The initial coding of the data was performed in accordance with Charmaz’ [21] description through line-by-line coding and identifying words or phrases related to the research question. This was done by closely studying fragments of data, words, lines, segments and incidents for their analytical importance. In the process of labelling the content of the data with codes, the following questions supported the process; “is this relevant for the study purpose?” “what does this data express?”, “what is the point of what is being said here?”, “is something left unspoken?” and “what is the meaning of the words expressed?”. Additionally, “action”, “meaning” and “process” were sensitising concepts used in the coding process (cf. [21]), which resulted in more action-driven codes; however, still close to the data.

**Fig. 1:**
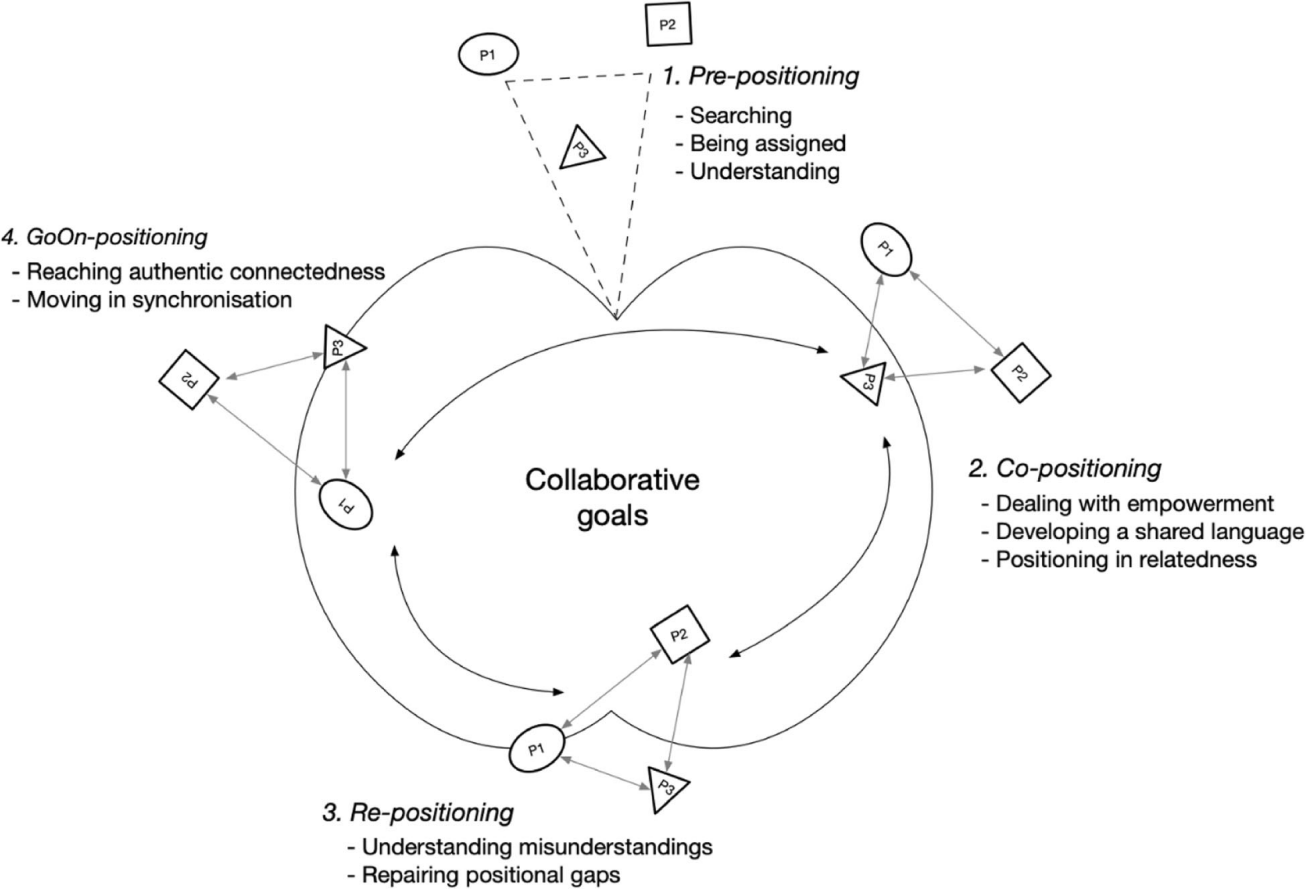
Conceptual model of Partnership Positioning. The process of partnership positioning consists of collaborative goals with four interrelated phases: (1) Pre-positioning, (2) Co-positioning, (3) Re-positioning and (4) GoOn-positioning with corresponding steps in each phase. In addition to the interrelationship between the four phases, interrelationships within and between the partner groups also exist. Additionally, the positions of the involved partner groups (p1, p2 and p3) are changeable through the process

The phase of initial coding was followed by the focused coding phase where the codes were selected, reflected on, further analysed, sorted and clustered. This was done by comparing the codes with other codes and deciding which would function best as focused codes. During this phase, analytical questions were used, such as “what do I see?”, “are the codes raised to focused codes?” and “do this/these code/codes have analytic power?”. The focused codes were clustered and transformed into categories. A form of theoretical coding was applied, which means studying the categories and identifying possible relationships between the categories. Developing theoretical sensitivity through theorizing included seeing and searching for possibilities, establishing connections and asking analytical questions. This included using theoretical playfulness with the purpose of being opened to the work and to ideas that best fits the data. The action-driven codes as well as discussions between the authors regarding possible relationships supported the theorizing since it was useful for seeing sequences, patterns and connections (cf. [21]). The dynamic relationships between the categories have been visualised in a conceptual model (Fig. 1). In the last step, the final categories were compared with existing theories [22] assessed as relevant and applicable to deepen the understanding of the findings.

As described above, with its numerous steps and phases, the process could be understood as a linear sequence; however, this was not the case. The process was indeed of an iterative nature where constant comparative methods were used. This included a constant movement back and forth between codes, categories and the text as well as comparing codes with codes, code/s with category/ies and categories with categories during the analytical phases (cf. [21]). Additionally, memo-writing was emphasised during all stages of the research. The memo-writing helped to simplify a deeper consideration and reflection throughout the whole process - from formulating the purpose, during the analysis process and when writing up the findings. Memo-writing was performed both chronologically in a separate memo document as well as during the analysis in direct connection with specific initial codes, focused codes and preliminary categories. The memos functioned as transitional steps between the analytical steps (cf. [21, 22]).

## Results

The core category *Partnership positioning* summarises the social process of the academic-community partnership. The grounded theory generated is presented through four categories: *Pre-positioning*, *Co-positioning*, *Re-positioning*, and *GoOn-positioning,* with corresponding subcategories.

### Partnership positioning

As a core category, Partnership Positioning covers the social processes of the academic-community partnership in a collaboration project to implement and evaluate health-promoting interventions in clinical health care. Positioning is conceptualised as a process that includes individual positioning as well as positioning in relation to others. Thus, navigation through one’s own and others’ beliefs, autonomy, roles and visions concludes the course of actions taken towards the construction of and intervention in a common ground in which connectedness can form the basis of well-functioning and future-oriented partnership. The process of partnership positioning consists of collaborative goals with four interrelated phases: (1) Pre-positioning, (2) Co-positioning, (3) Re-positioning and (4) GoOn-positioning with corresponding subcategories as visualised in Fig. 1.

In addition to the interrelationship between the four phases, interrelationships within and between the partner groups also exist. Pre-positioning is the first phase, and to a fairly large extent an individual process of grasping one’s own position in the partnership, while the following phases have a more dynamic interaction within and between the partnerships. Thus, the process of partnership positioning is not a linear process but is instead a continuing process with transfer between the phases. As visualised in Fig. 1, the positions of the partner groups (named as p1, p2 and p3) are not constant, meaning that they are changeable, and the positioning is a process and consists of actions dependent on and influenced by who has the power and capacity to lead the task. Collaborative goals are what drives the activity, and are therefore both a prerequisite and the driving force for partnership positioning. In short, collaborative goals in the present study cover the ambitions and visions for improving palliative care. Overall, the partnership has the strength to together construct a joint body of knowledge.

The journey of partnership positioning could be described as being initially merely individual parts or fragments that move towards the development of a togetherness and becoming partners. It is about understanding one’s own role, the roles of others, the relationship between roles, managing differences and obstacles, identifying and acknowledging strengths and resources within the partnership and having faith in the future. Consequently, the aim is to merge these perspectives into a mutual togetherness. Diversity in the organisational cultures of the partners had the possibility to combine unique perspectives and expertise. However, the cultural differences also posed some partnership challenges, for example when a mutual understanding of each other’s cultural context and beliefs was not reached, the acknowledgement of the differences and coming to an understanding was hindered. The partnership positioning was not always as even or symmetrical as the figure represents since inequalities in the teamwork emerged regarding the anchoring of the partnership, level of influence, and the possibilities for and quality of communication. Furthermore, at times there was a schism between a partner positioning oneself and being positioned by others.

### Pre-positioning

This category describes defining and identifying one’s tasks and roles in the partnership composition. It involves searching for tasks and roles, being assigned tasks and roles and developing understanding. Thus, the pre-positioning phase includes trying to find one’s position in the partnership construction, and likewise, being positioned by others.

#### Searching

As a subcategory, searching describes the actions taken towards finding one’s tasks and role in the partnership. Finding one’s role was seen as a process, from initial confusion towards a more solid and clear understanding. Challenges connected to searching for one’s tasks and role included difficulties in finding up-to-date information, such as progress plans and clearly specified division of responsibilities and assignment descriptions.

*So, I think the assignments might need to be a little more structured, like what it is supposed to be from the beginning, responsibilities, who does what.* (860178).

#### Being assigned

This subcategory involves range, and the means for being included in the project and the partnership. The acceptance and commitment to join and support the partnership varied between and within partner groups. From being caught by surprise with ambivalent feelings of involvement, to levels of high willingness for collaboration with dedication. Even feelings of stipulated inclusion emerged, when not having the opportunity to decide if one wanted to be included or not, i.e. being obliged to be included. A need to be involved earlier in the process as a part of the anchoring work was verbalised:

*It just came to the workplace that this is to be done and then maybe one did not have the big picture with the prior discussions about how this had developed.* (860178).

#### Understanding

This subcategory extends beyond merely finding one’s task and role towards having a comprehensive understanding of it. An understanding of the purpose of the overall task, an action to improve palliative care, was visualised. However, an understanding of the meaning and performance of separate instrumental tasks was not clear. Lack of clarity regarding the performance management process in combination with a lack of specific task and goal completion were revealed. One of the partner groups, especially, expressed confusion regarding comprehension of their tasks and roles. Feelings of loneliness, frustration, insecurity and, at times, embarrassment emerged when not being able to understand one’s role and task. Furthermore, it was challenging for one of the partner groups to support their staff in their assignments when they did not understand the big picture themselves.

*and I feel that I have been probably a little too little … a little too little informed to be able to push on. // Now I have kind of relied a bit on the fact that X has pulled the whole load because I know that she kind of has talked to the employees …* (860178).

### Co-positioning

Co-positioning was seen as a step after the pre-positioning phase where the positioning in relation to the other partners was visualised. The co-positioning phase extends beyond identifying one’s own role to understanding the role of others in relation to one’s own. It consists of handling autonomy and authority, developing a shared language and, subsequently, positioning in relatedness.

#### Dealing with empowerment

This subcategory includes hierarchal structures and mandates concerning aspects related to having the power to act, power over others and shared ownership. The experiences regarding addressing and dealing with ownership varied, involving a range of feelings from powerlessness to being in charge. Experiences of an uneven distribution of power between partners emerged, including experiences of being a limited partner, for instance, regarding conflict management in relation to power relationship. On the other hand, shared ownership with joint decision-making was emphasised and, furthermore, seen as a prerequisite for shared understanding. In addition, decisions based on the overall situation were presented, including the preferences of all partner groups.

*So, I thought it was very positive and we were so well treated in this and they actually pointed out in words that this is mutual.* (860155).

However, equal opportunities for influence and decision making within the partnership were not secured due to the line of authority. The decision-making process and mandate were not understood by all members, and moreover, they were not always shared or transparent. A variation from a symmetric to an asymmetric autonomy between the partnership groups emerged with fluctuating experiences of self-government and control.

*A little reverse gatekeeping. A bit like a position of power. What, what opportunity does one have to have an opinion if the boss tells you, instructs you, that you should join. These are interesting and important aspects to problematise, I think.* (860155).

Overall, one of the partners was experienced as the standard setter and functioned as an intermediary – a link between the partners. Furthermore, reflections of the level of closeness and co-determination emerged where the importance of preserved autonomy and freedom was highlighted. The leadership was viewed as competent and well-functioning when a balance between structure and freedom occurred, including freedom under responsibility. Discussions of ownership, including boundaries, to achieve a balance in authority were invited. Consequently, dealing with authority was a process, including experiences of positioning oneself in relation to power and ownership as well as being positioned by others.

#### Developing a shared language

This subcategory describes aspects of and actions in finding a common language that, in addition to verbal language, requires an understanding of each other’s organisations. There were some discrepancies in the verbal language when communicating between the partner groups. Having too dissimilar wording and tone between the partners when communicating resulted in not being able to comprehend each other and feelings of frustration and incompetence.

*I have been to a number of meetings where a manager has been completely exhausted and thrown the papers on the table // she explains that she has been to a research meeting and that she doesn’t understand anything. She just feels stupid.* (860150).

Within the partner groups, a verbal synchrony was experienced when the use of words and the understanding of concepts was congruous. However, in the verbal communication between the partner groups, some dissimilarities regarding use of concepts and understanding of the association of the words and concepts emerged. Even central concepts for the intervention (i.e. palliative care) were verbalised and understood in different lights. In order to strengthen the development of a shared language, reciprocal adjustments were taken with the intention of increasing awareness of how one’s words could be understood and interpreted by others, and consequently adapting the concepts and expressions used. For instance, the partners tried to avoid technical terms and replace them with more commonly used general words and concepts.

Furthermore, aspects emerged related to understanding each other’s organisations, function, composition and cultural aspects. The conception of an understanding of others’ positions in the partnership was connected to insights and understanding of other partners organisational contexts. This was central, since it contributed to an increased understanding of work, beliefs, values and strategies resulting in increased understanding of the decisions and actions taken, and also valued as a prerequisite for further co-construction.

*A project, it grows with time. And one gains more and more knowledge about each other in the different project groups. Our intentions. Their intentions. Our problems and their problems. So that’s how you do it, you develop the collaboration.* (860155).

#### Positioning in relatedness

This subcategory is about conveying and uniting perspectives. Positioning in relatedness extends beyond finding a shared language, subsequently including the process of the social positioning within the partnership. Relatedness positioning involves the understanding of one’s position in relation to others, i.e. the relationship between the different partners’ roles and tasks. This was viewed as fundamental since it affected the building of a solid foundation to value the demands of tasks, setting up plans, selecting suitable approaches and monitoring progress. It includes the view of individual expectations as well as organisational expectations concerning role-taking within the partnership.

The grasping of one’s position in relation to other’s varied within and between groups as well as over time. The process of discovering one’s own and others’ positions covered identifying one’s own role and subsequently making this role visible for others. Actions taken to facilitate the definition and comprehension of different positions and responsibilities of the partners included discussion and reflections with colleagues within the partner groups and, to some extent, between partner groups. The opportunities and means for developing this understanding varied. Partnership norms in the form of strategies regarding partnership interaction and communication were perceived as both being fulfilled or lacking. When communication was experienced as effective, it prevented misinterpretation and enabled the partners to keep up with the changes and adjustments that arose. A willingness to work together was expressed and, even when the covid-19 pandemic broke out and challenged the collaboration project and thus the partnership, the partners communicated and adjusted the planning and design based on the altered prerequisites of the different partners.

However, communication was viewed as asymmetric in that one of the partners was seen as the link between the other two partners meaning that the communication channels between the partners were not always straightforward and clear.

*So, we are three partners in this, but we have had more communication with one partner.* (860155).

Without regular and natural interaction and communication channels, the creation of an awareness of the others’ context was delayed and, at times, not achieved. Furthermore, it sometimes resulted in not being able to recognise or value the other partner’s contribution. A need for more symmetrical interaction was expressed to increase transparency and the development of a higher degree of mutuality with capacity to place oneself in another’s position. Moments where the partners positioned themselves closer occurred and were described as opportunities to share understanding within a reciprocal companionship that supported reconciliation of different organisational cultures and ways of working.

### Re-positioning

This category covers the process of renewed understanding of positioning where some previous positions were challenged, consequently resulting in re-positioning.

#### Understanding misunderstandings

This subcategory includes repositioning when reaching transformed understandings of one’s own and others’ positions concerning tasks, roles and power relations.

Some initial understandings were challenged regarding power distribution and mandate leading to an altered way of viewing ownership distribution.

*I thought from the beginning, that it is probably X who sort of owns this. But I have also come to get feedback as well and understand that it [the project] is shared.* (860155).

Re-positioning also includes taking on a new role and tasks from others and was especially difficult in the light of reorganisations and high staff turnover. When a person entered a position previously maintained by another this was challenging, since misunderstandings arose based on the confusion of taking on a poorly defined role. Thus, a need was raised for an enhanced structure as a routine to support new employees in the initial phase of understanding the project and the cooperation within the partnership. Entering the partnership at a later stage meant missing parts of the former phases of positioning (i.e. pre-positioning and co-positioning).

Even during the focus group discussions, when discussing understanding of roles, new insights concerning not understanding positioning arose leading to clarification of some previous misunderstandings. However, the communication channels and the continuity were not secured and a need for more forums to communicate and, thus enhance understanding, were acknowledged. The quality and quantity of communication were highlighted to be able to move from misunderstandings to clarity regarding both one’s own and others’ positions. For communication to be satisfactory and enable a movement from confusion to stability, both a sender and a recipient is required. Thus, within the dialogue – when talking *together* – the “right” positions to take became clearer:

*But, but, but I think it has sort of emerged, in the beginning the division of responsibilities was very unclear but in this dialogue between us it has become visible who does what.* (860155).

#### Repairing positional gaps

Through the process of positioning, some positional gaps emerged meaning that some roles and tasks had no clear holder, or at times several holders or overlapping positions. Some areas were viewed as a kind of no-man’s land, meaning tasks having no clear owner. Over time and through reflecting on where there seemed to be a positional task gap (i.e. lack of clarity of task holders) the areas of responsibility became clearer. As a result, some initial positions were at times challenged with subsequent re-positioning.

Perceived role compatibility played a central part in the re-positioning process where responsibility for different tasks was adjusted at times. For example, the members within one of the partner groups viewed their roles and responsibilities as being quite different and adjusted the role of responsibility by taking on either more or less tasks and by delegating some tasks to the staff. This was done in relation to the context at the specific nursing home, such as patient clientele, previous experience with academic collaboration, and range of access to nurses, palliative care agents and motivated and enthusiastic staff. The process of repairing positional gaps, and consequently re-positioning, was viewed as a process of trying, evaluating, and re-trying, or as one participant described it:

*And there has been this, what should I say, this openness and dialogue so that all the time we think like this, we try it out, we correct, we go back or like this works and this does not.* (550153).

### GoOn-positioning

GoOn-positioning is understood as a point of departure for a healthy, well-functioning and effective partnership collaboration. Reaching the phase of GoOn-positioning comprises being in an authentic connectedness within the partnership with subsequent coordinated movements towards the project goal. Consequently, it is about reaching a common ground guiding the partnership to reach its full potential.

#### Reaching authentic connectedness

This subcategory concerns aspects of true understanding, faith and respect in the togetherness of creating something greater than what could be achieved alone. Reaching authentic connectedness goes beyond relating to each other (i.e. the subcategory positioning in relatedness) meaning that connectedness was characterised by a trusting intertwined interaction; a connectedness with true mutual understanding and an acknowledgment of one’s own and the other partners’ context and contribution.

Achieving authentic connectedness was pictured as a journey where positioning was understood as individual perspectives intertwined with group specific perceptions with further development towards a solid partnership common ground.

*We had a conference call maybe two or three weeks ago //. And so, it was the first time I felt this real connection between the projects. That it was like bridged over a bit.* (860155).

However, as described in previous categories, some obstacles still had to be overcome in order to reach this authentic connectedness, since the partnership had mainly demarcated a joint overarching goal (i.e. improving palliative care) but, at this point, had not fully reached the stage of authentic unison. Furthermore, the transition, and thus level of reached stages of the partnership positioning, varied within the partner groups and between the partner groups. One of the partner groups, in particular, experienced not being fully taken into consideration.

*Eh a little more dialogue during this journey so you do not like eh, so you are on board.* (860178).

Nevertheless, a trusting relationship was under construction, where having faith in other partners’ strengths was acknowledged. For instance, the acknowledgement and value of the expertise within the different partner groups was expressed in all the partner groups. Uniting existing expertise and combining the specific professional skills and knowledge of their context were seen as an opportunity to learn from each other in constructing connectedness.

#### Moving in synchronisation

This subcategory represents a vision of the partners operating in an interplay where coordination of the partners’ actions operates as a system in harmony. This is a synchronisation where the partners’ different functions can be combined and act as a joint body of knowledge and skills where a type of synergy effect can be achieved. However, this was mainly described as a goal and not as a stage that had been reached.

Synchronised movement does not mean moving in exactly the same way with the same perspectives and specific goals, but rather the purpose is to combine goals, perspectives and expertise with the goal of true interplay in the way forward to a successful partnership project. It concludes with being responsible for different parts and, at the same time, being able to construct something greater than what can be achieved individually.

*Then I got this first feeling that: yes, we have actually reached a point in our dialogue. Everyone seems to understand the situation right now, how, where and what goals we have ahead. And how, who, and the division of responsibilities becomes very clear I think, who does what. Yes, you do that, I do that and we do that.* (860155).

Additionally, a future vision of continued cooperation was clearly verbalised as something valuable for both this project and for future collaboration projects.

## Discussion

The findings show that, as a process, academic-society partnership positioning develops through transitions. The process starts with the initial phase (pre-positioning) of searching, being included and understanding one’s tasks and roles, subsequently moving on towards positioning in relation to the other partners (co-positioning). When reaching some understanding of existing and, at times, misplaced or lacking positions, a renewed understanding of positioning emerges and positions were at times challenged resulting in altering positions (re-positioning). The final phase of the process, although not yet completely attained, consists of reaching common ground where an effective partnership collaboration in authentic connectedness within synchronised movement can reach its full capacity (GoOn-positioning). The transition through the phases was dependent on and developed through interpersonal processes where organisational cultures and contexts had to be identified and communicated in order to develop an understanding of differences and preconditions that could facilitate and challenge a successful partnership construction. Over time, based on their own or others’ experiences, mistakes and successes in the actions in the partnership shaped various conceptualisations of how to nurture the partnership positioning. This meant that the process of partnership positioning was not a linear process and aspects in each phase were addressed throughout the partnership. Consequently, steps, actions, views and positions in each of the phases need to be revisited and at times strengthened or adapted.

This study of partnership positioning explains what happens in a new collaborative partnership with participants from different healthcare settings and academies. Our findings illustrate that the journey of partnership positioning involves moving from initially being merely individual parts, or fragments, towards the development of togetherness and becoming partners. Partnership positioning is about understanding one’s own role, the roles of others, the relationship between roles, managing differences and obstacles, identifying and acknowledging the strengths and resources within the partnership and having faith in the future. The interpersonal encounters are in focus, and rights and duties are distributed among people in changing patterns as they engage in the implementation project. How the people in the team are and behave is partly constituted by what roles, rights and duties they have. Altogether this can be seen in the “Positioning theory”, which focuses on interpersonal interactions and the attributing of positions among those interacting. This theory can be applied to understand the interactions between people both at an individual level and at group level, where people serve as group representatives [23]. The term intergroup positioning means the positioning of oneself or others at an individual level based on group membership and the positioning of oneself or others at group level. In line with the positioning, the theory says that the meanings of people’s actions are social acts and such positions are constituted by their assigned, ascribed, claimed, or assumed rights and duties to make use of the available and relevant discursive tools (ibid).

Inequality in teamwork arose from the lack of anchoring of the partnership and lack of shared influence, opportunities and quality of communication. In addition, there was sometimes a split between how one positions oneself and how one is positioned by others. This is in line with Bronstein’s [24] model for interdisciplinary collaboration. Among one or more professionals from different disciplines engaged in work-related activities, these processes should represent five core components: a) interdependence, b) newly-created professional activities, c) flexibility, d) collective ownership of goals and e) reflection on processes. In our study, we saw barriers to collaboration if roles were not clarified. According to Bronstein (2003), to function interdependently, professionals must have a clear understanding of the distinction between their own role and the collaborating professionals’ roles. The participants want structure and try to understand what and how to do things. Shared ownership with joint decision-making is seen as a prerequisite for shared understanding. An early definition of collective ownership of goals also seems highly important since, at least from the beginning, it was interpreted that the ownership and the implementation had a top-down view.

The conception of understanding others’ positions in the partnership is connected to insights and understanding of other partners’ organisational contexts. Flexibility and reflection are also essential, not least related to creating an understanding of the function, composition, and different cultural aspects of each other’s organisations. Without regular and natural interaction and communication channels, the creation of an awareness of the other’s context was delayed and at times not achieved. Re-positioning also includes taking on a new role and tasks from others and was especially difficult in light of reorganisations and high staff turnover. This required flexibility and was challenging, since misunderstandings arose based on the confusion of taking over a role that was not well-defined and some roles and tasks had overlapping positions.

The findings in our study, especially regarding the GoOn-positioning phase, can also be understood through the lens of Sandberg’s [25] concept of collaborative health. Sandberg [25] describes *collaborative health* as a concept “synonymous with health aspects of co-operation within a working group of frequently interacting colleagues, such as a team”. The nature of collaborative health consists of *functional synergy,* which occurs when both sharp goal-orientation and the creation of synergy arise at the same time. Our study reveals that the partnership positioning was definitely in progress, even if it had not fully reached its full potential and the stage of GoOn-positioning with the authentic connectedness with subsequent coordinated actions. In addition to our previous reflections in this discussion, the time-aspect could play a role since, according to Lawson [26], collaboration takes years to develop and even longer to become institutionalised. However, collaboration is an essential element in innovation and solutions to large, unstructured, complex problems require vision, collaboration and innovation [27]. Hence, it is also of great value when planning a collaboration project to have a relevant time plan for collaborative health and functional partnership.

Taken together, the social processes concerning positioning in academic-community partnership consists of constructing a form of alliance that transpires in multiple forms and at different levels. In the light of the study findings, the cross-disciplinary and cross-organizational partnership addressed some potential cultural and system-level barriers in their transition towards forming a common ground with a mutual ambition of reaching full partnership effectiveness. To enable coordination and delivery of safe, high-quality care, reliable teamwork and collaboration within, as well as across, organisational boundaries is required [28]. A well-functioning academic-community partnership have the power to create sustainable relationships, improve community care [29] and support health equity [30, 31].

### Methodological considerations

Berthelsen et al. [32] describes the importance of presenting and explaining criteria for evaluating the quality of grounded theory, allowing the reader to assess trustworthiness. The criteria of trustworthiness described by Charmaz [21] are applied to discuss and present reflections and actions taken to bring about methodological strengths. According to Charmaz and Thornberg [22], C*redibility* includes having sufficient relevant data and making systematic comparisons throughout the research process. In this study, the perspectives of different collaborating partners were included, which is seen as a strength in the assessment of the social processes of partnership. Additionally, the data collected through four focus group discussions and one individual interview were rich in content with a variety of responses. The use of focus group discussions for data collection was experienced as favourable since it allowed group interactions and has the power to reveal experiences that might not have been possible without the group interactions (cf. [33]). The atmosphere during the focus group discussions was open and permissive, and no disputes or disagreements occurred. Regarding the interview guide, we strove to enhance its credibility by critically judging it, both independently and through joint reflections by SP and SL who also performed the focus group discussions.

Additionally, credibility includes the researcher’s views and actions. According to Charmaz [34], it is essential that the researchers reflect on their own methodological self-consciousness. From a constructivist perspective, we embrace the approach that we all have frames of reference where neutrality is impossible and not preferable. Thus, we assume that both data and analyses are social constructions that reflect what their production entails, meaning that we position ourselves subjectively in the construction and interpretation of data (cf. [21]). In the constructivist approach, our previous knowledge and experience that can be relevant to the study covers decades of clinical work as registered nurses, and years of clinical and research-related experience and knowledge in implementation, teamwork and implementation science. As authors, we have discussed our previous experience with the intention of using aspects of it to deepen understanding and interpretation without letting it take control. We have managed our preunderstanding by using reflexivity throughout the process. Dahlgren [35 p.128] figure of “the dynamic between open-minded interpretation and use of preunderstanding during the research process” has supported the handling of preunderstandings throughout. In broad terms, this meant that SP strove to be open to the data, (i.e. to be directed/influenced by preunderstanding to a lesser extent) during the initial coding phase. Later, the use of preunderstanding successively enhanced the next phases (ibid.). The reflective and interpretative stance in the interaction with the participants as well as during the analysis are further described and exemplified below in this methodological discussion (cf. [32]).

Furthermore, in relation to credibility, the analysis process is thoroughly described in the method section, including the challenges, different actions taken and the reasons for taking them, memo-writing, the use of sensitising concepts, constant comparisons and examples of questions asked of the data during analysis. Additionally, examples and quotes from the data demonstrate how the coding process developed and how the conceptual categories were formed from and grounded in the data. Regarding the findings, theoretical saturation was judged to have been reached since categories were assumed to be saturated because gathering and analysing the data from the last focus group discussion revealed no new theoretical insights of the emerging grounded theory (cf. [21]). Furthermore, all participants from the three partner groups had been invited to participate in the focus group discussions. However, to expand the understanding of partnership positioning, it may have been valuable to obtain the perceptions of the healthcare professionals (i.e. registered nurses, enrolled nurses, care assistants, physiotherapists and occupational therapist) who are actually delivering health and social care. Thus, we encourage such a focus and view it as an implication for further research.

As part of the study linked to credibility, member-checking was applied where SP and SL invited all the participants to digital discussions of the findings. A total of four sessions of 45 min each were offered to participants who could decide whether they wanted to participate and, if so, which session they preferred. In total, nine participants participated in one of the offered member-check sessions. In addition to these discussion sessions, the participants had the possibility to leave anonymous feedback via an online document. In general, the participants expressed that they recognised their discussions in the presentation of the findings and perceived that the findings were based on their own role, for example that of a researcher or a manager at a nursing home. However, in some of the member-check discussions, the participants did not recognise all the content, suggesting that this may be because some time had passed since the project was conducted and that they had participated in different focus groups during the discussion. The GoOn-position was mentioned as not being an end point, but rather the beginning and/or a continuation of a movement. Based on the member-check feedback, the authors revised some wording of certain terms and concepts, and the conceptual model of Partnership positioning (Fig. 1) was re-designed to more clearly visualise the dynamics between the phases.

Regarding *originality,* the findings of this study offer new insights into the construction of a common ground in partnership (cf. [21]). The use of the theories and models regarding interdisciplinary collaboration and systemic thinking improves originality in terms of a deeper understanding of the phenomenon. A strength regarding *resonance* is that the constructed categories and concepts represent both the participants’ experiences of partnership as well as providing insights into the dynamic social process of academic-community partnerships (cf. [22]). The findings of this study link to *usefulness* since the final model can be used in practice to support successful development of academic-community collaboration. The model can be used to enhance understanding of aspects affecting partnership and consequently support partnerships in constructing a common ground. Another aspect related to usefulness is *transferability* [36, 37], which refers to if and how the findings can be transferred to other contexts. The authors have given suggestions regarding how the findings can be used, nevertheless it is up to the reader to judge and decide if and how the findings can be applicable in other contexts.

## Conclusions

Partnership positioning is about entering into something unknown; a new role in a new culture, combining one’s own perspectives and beliefs with that of others, overcoming feelings of inadequacy, and moving towards an overall common ground with shared goals that benefit the patients. Collaboration in teams in health care can take many forms and teamwork takes place in several different contexts. The common denominator is that different competencies complement each other to achieve the best results and that collaboration in teams is a key factor for person-centred and patient-safe healthcare. Workers in healthcare settings have always been expected to collaborate [24]. However, our findings indicate that a new partnership in an implementation project is complex and needs holistic, systemic thinking. Hence, a plan is required to enhance implementation when planning a collaborative project with different professionals and actors, and to facilitate positioning activities. The process, the roles and the components need to be clearly defined and documented, and the management of a system requires knowledge of the interrelationships between all the components within the system. A wide range of communication opportunities is needed for the actions to be understood and processed by the partners. It is also essential that the different team members work together and support each other to perform as a whole.

Teamwork is an established way of working in health care and is also a central core competency. Another important aspect of high-quality health care is the need for constant healthcare development, since new knowledge and changing conditions in society must be met by the healthcare system. It is, therefore, important to enable new research findings to be translated into clinical work. Consequently, the improvement of knowledge with the goal of offering the best possible care is needed (cf. [38]).

### Relevance to clinical practice

Implementation of new knowledge often requires a multi-disciplinary approach, and these improvement processes can be time-consuming and complex. Participating in non-established teamwork – in this case an academic-community partnership– with the purpose of developing health care has challenges but also the strength to create common ground for working together towards the mutual goal of increasing the quality of health care. Creating a collaborative culture requires effort. Interactions in terms of role seeking and positioning have been shown to be of importance in many contexts when people come together, not least in interdisciplinary collaboration in health care. Joining a new collaborative project with a non-established team has challenges; professional roles, individual preferences, mutual concerns, and mutual decisions must be constantly defined, resolved and overcome. Consequently, to support the partnership positioning, collaborative activities and flexibility during the process are required. Collective ownership of goals, the roles, and the components in the intervention need to be clearly clarified early in the process.

## Data Availability

The datasets generated and analysed (i.e., transcribed interviews) during the current study are not publicly available due to the raw data containing information that could compromise the privacy of the research participants and due to the fact that participants of this study did not agree for their data to be shared publicly, but are available from the corresponding author upon reasonable request.
